# Progress

**Published:** 1884-07

**Authors:** J. B. Chandler


### PROGRESS.

J. B. CHANDLER.

The trait most prominent in the citizens of this great Republic, and the one which has most aided in the speedy development of the immense resources of the Continent, is progressiveness; the pressing onward; restlessness under anything short of perfection; study of causes and effects, and the adoption of the useful in the rejection of the useless.

The constant attrition of active brains has resulted, not only in wonderful and useful discoveries and ingenious adaptations, but also in raising the standard of the human brain itself, amongst us.

Man has advanced in his own capabilities; children now are starting far ahead in the race of life of those of the generation which preceded them. I do not refer here to education or what are known as acquired talents; I allude to the human base, the improved capabilities of the soil; the slow but sure manifestation of the natural result of "the survival of the fittest."

It is hard to trace the inherited attributes which the child receives from the parent; those which are alone the result of study and experience of the father or mother, and which are transmitted in the mystery of birth—not taught. That such attributes exist can be proven by the removal at a tender age of the infant from the parents and watching carefully the tendencies which indicate parental influence. Let us consider this trait or advancement as met with in other walks of creation and known popularly as "instinct." Are animals and birds educated by being brought in contact with men, or do they remain in the same rut they started in, never progressing?

The strings of the first net which caught crows did not forewarn the uneducated thieves; but those which escaped profited by the lesson, and the race have now become so cautious that the farmer finds a few poles with white strings connecting, protect the corn field. Has this been transmitted by oral education? "My little fledglings never trust strings around a field," or has this knowledge become engrafted on the instinct stock of the crow family and young crows advanced at the start beyond those that had not stolen corn under difficulties? Will the day come when the "fittest" will discover the cheat of the strings, and so advance still further in the mental or instinct scale?

The crow illustration will come home to every farmer's boy, but I have testimony gathered this Spring to give to a *supposed* advancement in this directon which is newer and will be probably less generally observed. Whatever credit may attach to an average expertness in fly-fishing, my friends amongst the disciples of Izaak Walton have accorded me, yet I should attribute what I am about to state more to failing sight, less promptness of simultaneous action between eye, brain and arm, than to advanced cunning in the trout, were it not that on comparison of experience with good fishermen upon the same streams this Spring, we all agree that out of a large number of "rises," the proportionate catch has been extraordinarily small. The tempting artificial fly is tried *before* it is taken in the mouth; the trout approach and detect the cheat, or jump out and fall on it; many are taken by the tail by the trailing fly whilst examining the upper "bobber." This is the result of observation carefully made on a closely-fished stream and tested by the experience of others. Are trout progressing?

Of course the human brain does not remain unequal to the emergency, and many who claim to be true disciples of old Izaak Walton have departed far enough from his teachings to arm the fly hook with something real, to appeal to other senses than the eye, and the slaughter of the innocents goes on.

"The fools are not all dead yet." Crows are yet caught in nets, and trout reward the patient cunning of the honest fly fisherman. If the fittest are to survive, the others must be sacrificed.

"Fittest" applies more to physical and mental abilities than to moral qualities, and with brain development comes not only ability for good, but, unfortunateiy, advanced talents for cunning and crime. Too prominently during the period since the last issue of the BISTOURY have the manifestations of the operations of the fittest for cunning and crime come to the surface. Many who had gone into Wall street prepared, as they thought, against all the known devices used there, have met their ruin, caught by new baits, resulting from the attrition of bright but unscrupulous brains, which have proven themselves fitter even for the work than their experienced coadjutors in the nefarious practices of stock gambling. This is progress in the wrong direction. A convention has been held at Chicago by what were supposed to be the representatives of the great political party which has controlled the destinies of this country during the past twenty-four years; two candidates out of a number seeking the places of President and Vice President have been nominated. The friends of the disappointed claim advanced cunning and other matters upon which it is not the province of the BISTOURY to touch, as influencing these nominations. Many influential newspapers point to this as a step towards progression, a step tending toward the correction of great errors and the survival of the fittest in the decadence of the corrupt. Others are jubilant because for once a man of undoubted ability, of high social standing, and with wealth beyond the need of emoluments from office has been nominated for the highest position in the gift of the people.

"The mills of the gods work slowly;" before we can mark this as a step in progress we must await results.

Another convention of a party which has easily earned for itself during the last two decades a "cap and bells," is to be held in July. Whilst I write this, many surmises are being made as to what that convention will do; they have a splendid chance for progress in both directions. They have not quite yet reached the *Ultima Thule* of folly. There is a little room on that road yet for progress, and they may occupy it; or they may astonish themselves and the world by a successful effort in the right direction which will place them in the company of the fittest.

Let these results be what they may, the stream of human life flows on; the great purposes of existence are being accomplished, and whether parties are retrograding or advancing, governments decaying or developing new greatness and power, men tending toward good or evil, natural laws are inexorable; the fittest will survive uutil the end shall come.

In the general dissemination of knowledge and power, people have become more confident in their own powers, and less willing to follow. Thus progress is too often made in the wrong direction, and not until the results prove disastrous will an effort be made to retrace or cut across into the right path, and even these efforts are too often so radical or intemperate as to impede the progress toward right which they contemplate, and result in renewed error; but fortunately, however, the agitation produces ultimate success.

We have amongst us men of great ability, who have naturally an attractiveness for, an control over, a large number of followers; such men possess enormous power for good or evil, and, unfortunately, some of them use that power for unscrupulous self-aggrandisement and advancement. But the fools who follow such are not increasing in number, and perhaps the time is near when the false fly will allure no more.

Let us, each individual, whilst carrying out the natural instincts of our peculiar race, encouraging progress, bear in mind that upon us rests a responsibility and the power to assist in directing that progress in channels which will tend to increase the happiness and comfort of those who are to succeed us.

